# A dynamical systems model for the measurement of cellular senescence

**DOI:** 10.1098/rsif.2019.0311

**Published:** 2019-10-09

**Authors:** Daniel Galvis, Darren Walsh, Lorna W. Harries, Eva Latorre, James Rankin

**Affiliations:** 1 Living Systems Institute, University of Exeter, Exeter, UK; 2 Translational Research Exchange at Exeter, University of Exeter, Exeter, UK; 3 Institute of Biomedical and Clinical Science, University of Exeter, Medical School, RILD Building, Barrack Road, Exeter EX2 5DW, UK; 4 Department of Biochemistry and Molecular and Cell Biology, University of Zaragoza, Zaragoza, Spain; 5 Department of Mathematics, College of Engineering, Mathematics and Physical Sciences, University of Exeter, Harrison Building, North Park Road, Exeter EX4 4QF, UK; 6 EPSRC Centre for Predictive Modelling in Healthcare, University of Exeter, Exeter EX4 4QJ, UK

**Keywords:** fibroblast, ageing, modelling, dynamical systems model

## Abstract

Senescent cells provide a good *in vitro* model to study ageing. However, cultures of ‘senescent’ cells consist of a mix of cell subtypes (proliferative, senescent, growth-arrested and apoptotic). Determining the proportion of senescent cells is crucial for studying ageing and developing new anti-degenerative therapies. Commonly used markers such as doubling population, senescence-associated β-galactosidase, Ki-67, γH2AX and TUNEL assays capture diverse and overlapping cellular populations and are not purely specific to senescence. A newly developed dynamical systems model follows the transition of an initial culture to senescence tracking population doubling, and the proportion of cells in proliferating, growth-arrested, apoptotic and senescent states. Our model provides a parsimonious description of transitions between these states accruing towards a predominantly senescent population. Using a genetic algorithm, these model parameters are well constrained by an *in vitro* human primary fibroblast dataset recording five markers at 16 time points. The computational model accurately fits to the data and translates these joint markers into the first complete description of the proportion of cells in different states over the lifetime. The high temporal resolution of the dataset demonstrates the efficacy of strategies for reconstructing the trajectory towards replicative senescence with a minimal number of experimental recordings.

## 1. Introduction

Ageing is a critical economic, social and medical challenge and understanding the underlying molecular mechanisms is becoming increasingly important. A major driver of ageing phenotypes in man and other species is the accumulation of a population of terminally growth-arrested but metabolically active cells termed senescent cells [1]. Gradual accumulation of senescent cells over the life-course contributes to tissue degeneration and age-related disease [2,3]. Over the past few years, removal of senescent cells has been demonstrated not only to delay the onset of age-related disorders [4] but also to bring about improvements in phenotypes as diverse as osteoarthritis [5], cognitive function [6] renal function [7], hepatic steatosis [8], metabolic function and adipogenesis [9] and vasomotor dysfunction [10]. Besides the cell cycle arrest, senescent cells show morphological, biochemical and functional changes such as secretion of the senescence-associated secretory phenotype (SASP) and high levels of DNA damage [11,12]. Accordingly, systems to remove or ameliorate the effects of senescent cells are currently the subject of intensive study.

Senescent fibroblasts generated by continuous culture until growth arrest (replicative senescence) provide a good model to study ageing and age-related disease, exhibiting many of the established hallmarks of ageing [13] and have been used to evaluate interventions designed to remove senescent cells [14–16]. Cultures of aged cells are a heterogeneous population, consisting of proliferative, senescent, growth arrested and apoptotic cell populations [17]. Tissues and organs in older humans are likewise a complex mixture of senescent, proliferative and growth arrested cell populations [18]. Determining the trajectory towards replicative senescence while distinguishing between senescence and reversible growth arrest is crucial for evaluating the effectiveness of new approaches to remove or rejuvenate senescent cells. Commonly used senescence markers are diverse and with none purely specific for senescence [19] meaning that accurate quantification of senescent cell numbers in different tissue types is still a challenge. Combining multiple experimental markers to better understand the transition of a cell population to replicative senescence is labour intensive and time consuming. Cells in a mixed population may be in one of four potential states: proliferating (viable cells growing through mitosis), senescent (irreversibly growth-arrested), quiescent (reversibly growth-arrested) or apoptotic (initiating cell death) [20,21].

The most frequently used marker for cellular senescence is senescence-associated beta galactosidase (SA-β-Gal) [22], although others have suggested the combination of the proliferation markers Ki-67 and PCNA, and the DNA damage marker γH2AX is optimal [23]. The use of single markers can however be problematic. Ki-67 for example can reliably detect cells in the G1 phase of the cell cycle, but can also positively stain cells arrested in G2 [24]. γH2AX will identify any DNA damage, not only that associated with senescence. Concurrent measurements of multiple markers throughout the replicative span therefore provide a more complete picture of the trajectory towards replicative senescence but require integration to properly interpret the proportion of cells in each of these four states during the course of senescence. There is a need for a deeper theoretical understanding (through a systems biology approach using computational biology) of the trajectory of replicative cell populations. A systems biology approach can be used to bring together biological knowledge, experimental evidence and sets of assumptions to simulate the replicative life-course of cell populations. Others have used dynamical systems modelling to model the proliferating and senescent populations using differential equations and were able to reproduce some of the characteristics of experimental markers such as population doublings and SA-β-Gal (similarly SAHF) but not of other markers such as γH2AX and Ki-67 [23]. Although promising, this approach has not been fully exploited for investigating cellular senescence.

**Figure 1.**
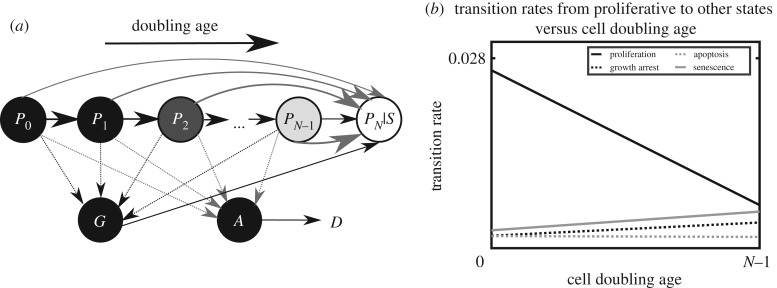
(*a*) Schematic for dynamical systems model of transition to senescence. Each proliferative state is represented by a population *P_i_* with doubling age *i* (to a maximum age *i* = *N*, the Hayflick limit). The transition of one cell from a population *P_i_* results in two cells entering the next population $P_{i+1}$. At any doubling age, a cell can jump to the senescent population *S* as a result of levels of DNA damage or other cellular stressor. Cells can also transition into the arrested *G* and apoptotic *A* states. (*b*) Optimized transition parameters. The transition rates from proliferative cells into other states are assumed to have linear dependence on doubling age. Linear relationships (slope and intercept) were determined through optimization against five markers (doubling population, SA-β-gal, Ki-67, γH2AX and TUNEL; see figure 3). The proliferation rates decrease with cell doubling age, reflecting the slowing of movement through the cell cycle as the doubling age increases. The rates of transition into senescence, arrest and apoptosis all increase with doubling age. The increasing rate of transition into senescence and apoptosis reflects the increased probability of these transitions due to factors such as DNA damage, telomere shortening and double-stranded breaks. The increasing rate of transition into quiescence reflects the effect of, for example, signalling from senescent cells.

We present here a dynamical systems model that captures the dynamics of four senescence markers (SA-β-Gal, Ki-67, γH2AX and TUNEL) over 16 passages in human primary fibroblasts to assess the trajectories of proliferating (*P*), senescent (*S*), growth-arrested (*G*) and apoptotic (*A*) cells over the replicative life-course. The model incorporates growth-arrested and apoptotic populations while also tracking the doubling age of proliferating cells. Parameters in the model control the rates at which cells transition between these different states (see schematic diagram of possible transitions in figure 1*a*). The doubling age of proliferating cells is modelled allowing for these rates to depend on cell doubling age (figure 1*b*). Genetic parameter optimization methods are used to set the model's minimal set of parameters for the model outputs to best match the recorded experimental markers. Once fitted to experimental data, the model simultaneously captures the dynamics features of all five experimental markers and, importantly, reproduces the proportion of cells in each state over the replicative time-course.

Moving beyond the state of the art, our work demonstrates that a simple set of rules governing transitions between cell subtypes can, when implemented in a dynamical model, capture the dynamics of cell proliferation over the replicative life-course. The model description is minimal, designed with a core set of parameters that are well constrained by our experimental data. To illustrate the model's utility in supporting experiments on senescence, an optimal strategy for the sparse collection of data is presented.

Our model provides a full account of the proportion of cells in each of four different states (proliferating, senescent, growth-arrested and apoptotic) and predicts the convergence of these proportions beyond the end of the experiment. It provides a valuable tool for measuring senescence and, therefore, for evaluating approaches to modify or reverse cellular ageing.

## 2. Methodology

### 2.1. Experiments on human primary fibroblasts

Human primary dermal fibroblast (nHDF) cultures were used in this study. Standard culture conditions were a seeding density of 6 × 10^4^ cells cm^−2^ in media (C-23020, Promocell, Heidelberg, Germany) containing 1% penicillin and streptomycin, and a fibroblast-specific supplement mix consisting of fetal calf serum (3% v/v), recombinant fibroblast growth factor (1 ng ml^−1^) and recombinant human insulin (5 µg ml^−1^) (Promocell, Heidelberg, Germany). Cells were counted, and equal numbers of cells seeded in 25 cm^2^ flasks at each passage in continuous culture. Cells were maintained at 37°C and 5% CO_2_ and senescence markers were measured in triplicate in each passage from p5 to p20. The biochemical senescence marker SA-β-Gal was assessed using a commercial kit (Sigma Aldrich, UK) according to manufacturer's instructions. Terminal DNA breakpoints *in situ* 3-hydroxy end labelling (TUNEL) was used to quantify levels of apoptosis. The TUNEL assay was performed with Click-iT^®^ TUNEL Alexa Fluor^®^ 488 Imaging Assay kit (Thermofisher, UK) following the manufacturer's instructions. Negative and positive controls were also performed. Cells were double stained for proliferation rate and DNA damage markers; Ki-67 and γH2AX were used respectively. Cells were fixed for 10 min with 4% PFA and permeabilized with 0.025% Triton and 10% serum in PBS for 1 h. Cells were then incubated with a rabbit monoclonal anti-Ki-67 antibody (ab16667, Abcam, UK) at 1 : 250 and a mouse monoclonal anti-γH2AX (ab26350, Abcam, UK) at 1 : 500 overnight at 4°C followed by FITC-conjugated secondary goat anti-rabbit (ab16667) and anti-mouse (ab150117) at 1 : 200 for 1 h, and nuclei were counterstained with DAPI.

### 2.2. Dynamics systems model to reconstruct the senescence time-course

The model captures the unconstrained growth of a cellular population during its transition from an exponential growth phase to a senescent state (no longer dividing), which happens between passage 5 (PD5) and passage 20 (PD22) in our experimental dataset. Individual cells within the model are classified as belonging to either a proliferative (*P*), growth-arrested (*G*), apoptotic (*A*) or senescent (*S*) population; see the schematic diagram in figure 1*a*. Cells within the proliferative population are further divided into proliferative subpopulations with respect to the number of times they are predicted to have divided. The subpopulation *P_i_* consists of cells that have divided *i* times. We call the index i the *doubling age* of the subpopulation (capturing e.g. the gradual degradation of telomeres following sequential cell division). Tracking doubling age in this way allows for the rates of transitions into other states to depend on it. There is a total of *N* proliferative populations, where *N* is maximum number of times a cell can divide prior to reaching the senescence state. Here, modelling nHDFs, we chose *N* = 50 and found that our results were not sensitive to this choice (discussed further below).

### 2.3. Transitions between cellular populations

The proportion of cells in each population varies over time as captured by a system of coupled differential equations. These equations describe the transition of cells between cellular populations over time. A full description of the equations is given in electronic supplementary material, while a summary of the assumptions used to define the equations is given here. We assume that at the beginning of the experiment the initial population of seed cells reside in the *P*_0_ subpopulation. The transition from one proliferative subpopulation to the next captures mitosis and so cells enter $P_{i+1}$ at twice the rate that they leave *P_i_* (shown as horizontal arrows in figure 1*a*). Cells can reach the subpopulation *S* by two mechanisms, the first of which is division. Since cells can transition to senescence by division of the population $P_{N-1}$, we sometimes use $P_{N}$ to denote the senescent population *S* (figure 1*a*)*.* Cells can also enter the senescent population directly from any proliferative population, with a rate that increases with doubling age (top grey arrows). This mimics a direct accrual of DNA damage, independent of cell division, which is sufficient to halt cellular proliferation. Thus, cells ‘jumping’ from proliferative to senescence enter the senescent population at the same rate they leave *P_i_*. Likewise, cells from all proliferative populations can enter growth-arrested and apoptotic cellular populations at the same rate they leave *P_i_* (arrows down to *G* and *A*). An example equation describing the rate of change of proliferative population *P_i_* per unit time is

$$\frac{\text{d}P_{i}}{\text{d}t}=2m_{P_{i-1}P_{i}}P_{i-1}-(m_{P_{i}P_{i+1}}+\;m_{P_{i}S}+m_{P_{i}A}+m_{P_{i}G})P_{i},$$

where the left of the equation expresses the rate of change of the number of cells in *P_i_*, which is proportional to the right side of the equation. In this equation the number of cells entering *P_i_* is proportional to $P_{i-1}$ and the number of cells leaving is proportional to *P_i_*. The rates described above are *m*, with a subscript for each population or subpopulation involved in a given transition; e.g. $m_{P_{i}S}$ is the rate of transition from the proliferating subpopulation *P_i_* to the senescent population *S*. Similar equations describe the rate of change of the number of cells for each proliferating subpopulation *P_i_* and each population *G*, *A* and *S*.

The system of equations can be solved over time (a simulation) to produce trajectories for the number of cells in each population, which can be compared to our experimental data. The rates *m* are assumed to depend linearly on the doubling age of the proliferating cells. These linear dependencies (figure 1*b*) are determined through an optimization routine as described below. The model parameters that determined the rates *m* are listed in table 1.

**Table 1.** *N* = 50 is the maximum number of times a cell in the population can divide (analogous to Hayflick number). The choice of *N*-value had only a minimal effect on the data fit (see electronic supplementary material). The transition rate parameters $m_{GS}$ and$\;m_{AD}$ are positive numbers determined through optimization (2 of 14 free parameters). The transition rate parameters $m_{P_{i}P_{i+1}}$,$\;m_{P_{i}S}$, $\;m_{P_{i}G}$, $\;m_{P_{i}A}$ are lists of *N* values with a linear relationship to *N* as set by the positive-valued endpoints $m_{P_{0}X}$ and $m_{P_{N-1}X}$ determined by optimization (8 of 14 parameters). The fraction of contribution from each population (*P* or *G*) to each marker (γH2AX or Ki-67) is set by $P_{\gamma H2AX}$, $G_{Ki-67}$, $G_{\gamma H2AX}$, which take values between 0 and 1, determined by optimization (3 of 14 free parameters). These contributions are not known but form part of the optimization cost function (rather than part of the dynamical system).

parameters	$m_{P_{0}P_{1}}$	$m_{P_{N-1}P_{N}}$	$m_{P_{0}G}$	$m_{P_{N-1}G}$	$m_{P_{0}S}$	$m_{P_{N-1}S}$	$m_{P_{0}A}$	$m_{P_{N-1}A}$	$m_{GS}$	$m_{AD}$
rates (h^−1^)	0.0259	0.0063	0.0018	0.0037	0.0026	0.0053	0.0018	0.0016	0.0007	0.0010
parameters	$P_{\gamma H2AX}$	$G_{Ki-67}$	$G_{\gamma H2AX}$							
proportions	0.2385	0.9979	0.8237							
parameters	*N*									
value	50									

The relationships between the model outputs and our experimental markers need to be defined in order to make a direct comparison. As our model continuously monitors the number of cells in each population, comparing the population doublings in the model to those measured experimentally is straightforward. For both we used the formula

$$\text{PD(}t)=\log_{2}\frac{\text{TP}(t)}{\text{TP(}0)},$$

where PD is the total number of population doublings and TP is the total population. In the model TP = *P* + *G* + *S* + *A*.

The output from the model's differential equations is the proportion of cells in each population *P*, *G*, *A* and *S*. In order to compare these with our experimental markers it was necessary to define relationships between the proportion of cells in each population and the proportion of cells of each type expressing the markers SA-β-Gal, Ki-67, γH2AX and TUNEL as measured in the experiments. It is assumed that SA-β-Gal and TUNEL map directly onto senescent (SA-β-Gal) and apoptotic (TUNEL) populations. However, faithfully accounting for positive Ki-67 or γH2AX staining in our model is more complex. There is evidence that Ki-67 is identifiable not only in proliferating cells, but also in a proportion of cells which are growth-arrested [25]. As such, as well as the proliferative population, Ki-67 also maps onto a fraction of the growth-arrested population within our model. Likewise the expression of γH2AX was assumed to be proportional to the entire senescent, entire apoptotic and fractions of the growth-arrested and proliferative populations (in part based on the observations that around 30% of cells expressed γH2AX at passage 5, when cells are predominantly proliferative, and close to 100% of cells expressed γH2AX at passage 20 when cells are predominantly a mix of senescent and growth-arrested) [26]. In each case the fractions were determined through optimization.

The model was fitted to the experimental dataset using a genetic algorithm in the MATLAB programming environment. A cost function was defined as the total difference between the experimentally recorded markers and those predicted by the model, as dependent on a set of 14 model parameters (table 1). A gradient descent routine was also used to further refine the optimization and verify that a local minimum of the cost function had been found. For full details of the optimization of the model refer to the electronic supplementary material.

## 3. Results

**Figure 2.**
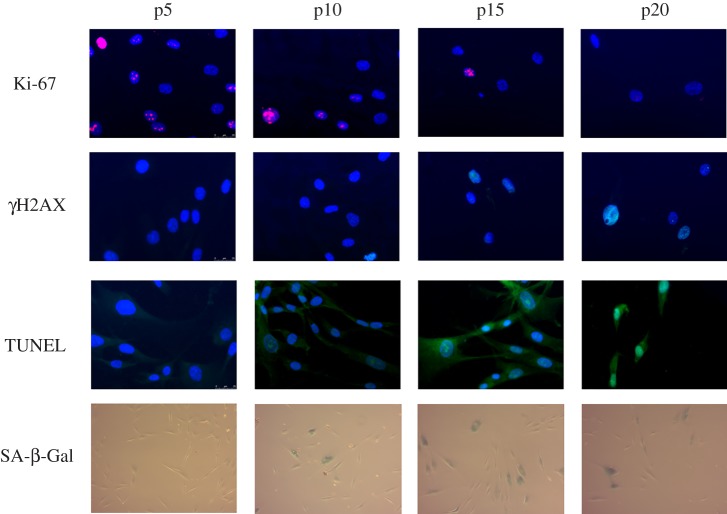
Sample images used for quantification of experimental markers of senescence in human dermal fibroblasts from an early passage (p5) to senescence (p20). For of Ki-67, γH2AX and TUNEL a blue fluorescing DAPI marker binding to DNA is expressed in all cells and an additional colour is expressed if the cell is positive for the given marker. In general the total number of cells decreases with time (note fewer cells at p15 and p20). For SA-β-Gal, cells expressing the marker are determined with a cytochemical assay that stains blue, while total cell counts are determined with high contrast images (not shown). Over time the proportion (percentage of total) cells expressing Ki-67 decreases (pink), γH2AX increases (light blue), TUNEL increases (green) and SA-β-Gal increases (blue). The markers were quantified by microscopy (using many images similar to those shown here), calculating the percentage of stained positive with a minimum of 300 cells assessed per replicate. Each marker was assessed in three biological samples per passage with two technical replicates. An automated haemocytometer was used for cell counts in order to compute population doublings (not shown). (Online version in colour.)

The following senescence markers were used in this study [27]: SA-β-Gal activity (typical enzymatic activity for senescent cells), Ki-67 staining (commonly used in the detection of proliferating cells), γH2AX assay (a marker of DNA damage) and TUNEL (detects apoptotic DNA fragmentation); see figure 2. We also assessed the population doublings (PD) to measure cell growth kinetics. The experimental results provide a percentage of cells expressing each marker, which can be compared with our model that tracks the proportion of cells in different states. While each marker is related to the proportion of senescent cells in the cell population they map to other cellular states including proliferating, growth arrested and apoptotic. The contribution of each distinct cellular population within our model (P, G, S and A) to each experimental marker was defined (see Methodology for details), allowing direct comparison of experimental and model cellular populations along the trajectory towards senescence. The experimental data were then used to constrain the model we develop via parameter optimization.

### 3.1. Model accurately captures five experimental markers

**Figure 3.**
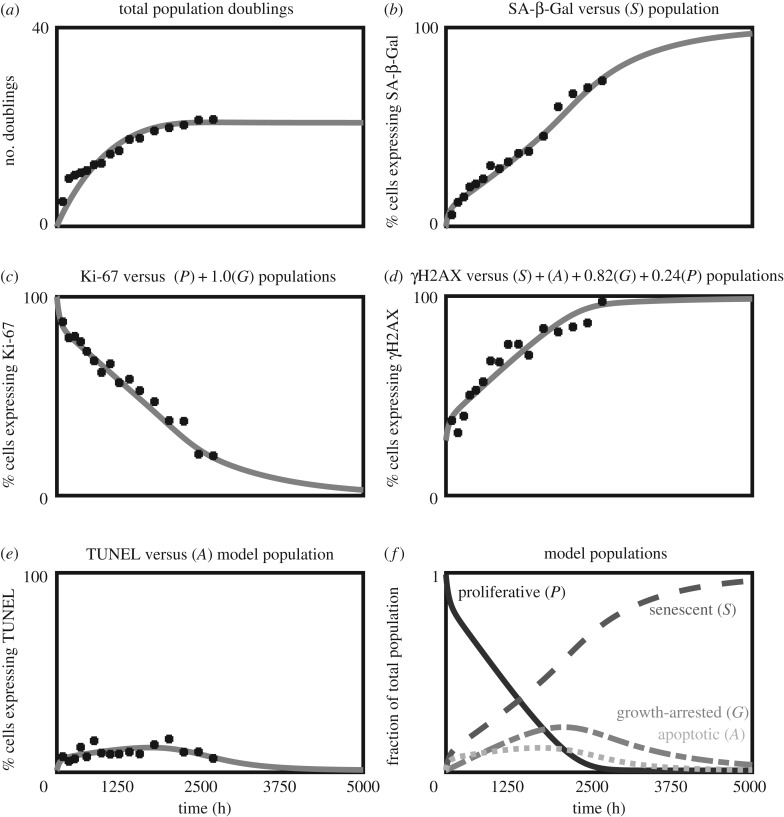
Optimized model trajectories versus markers. Experimental data points for human primary fibroblast cells recorded from passage 5 (PD5) to passage 20 (PD22) plotted with time in hours: PD (*a*), SA-β-Gal (*b*), Ki-67 (*c*), γH2AX (*d*), and TUNEL (*e*). The best fit trajectories from the dynamical systems model are plotted as grey lines. In the case of Ki-67 (*c*), where some unknown proportion of growth-arrested cells are represented, we include the proportion given by the optimizer in the panel title. Likewise, in the case of γH2AX (*d*), where an unknown proportion of growth-arrested and proliferative cells is represented, we include the proportion given by the optimizer. Panel (*f*) shows the complete senescence time-course for each population over 5000 h. As expected, the proportion of proliferative cells starts near 100% and decreases to 0% over the length of the experiment. At the end of the experiment, only senescent and growth-arrested cells remain.

Our results agree with the trajectory for these markers reported in previous studies [28]; see figure 3*a–e*. By passage 20 (PD22) (around 2500 h of cultivation) the cell population growth has plateaued; see figure 3*a* where the last two points have similar values. The expression of SA-β-Gal increases steadily over time reflecting the larger proportion of senescent cells as the population ages; see figure 3*b*. There is a similar regular increase for γH2AX primarily due to the accrual of DNA damage; compare figures 3*b* and 3*d*. The proportion of proliferative cells decreases with time as reflected by the consistent decreases of Ki-67; see figure 3*c*. The proportion of apoptotic cells at the end of each passage remains relatively steady throughout the experiment as reflected by the TUNEL measurements in figure 3*e*. This dataset of 16 time points across five makers provides ample data to constrain the model's 10 free parameters.

Following parameter optimization, the model (grey curves) was able to simultaneously capture the features of all the trajectories of all five markers (black points) from the experimental dataset; see figure 3*a–e*. The model's dynamic variables directly capture the proportion of cells in each state, thus revealing the trajectories of each population over the senescence time course; figure 3*f*. Note that the model's representation of each marker in figure 3*b*–*e* (grey curves) is assumed to be proportional to one or more of the populations *P*, *S*, *G* and *A* with constant scaling factors as indicated in the panel titles.

The trajectories of the different cell populations shown in figure 3*f* show that for early passages (approximately time 100–2500), the proportion of proliferating cells (*P*) decreases linearly, while the proportion of senescent cells *S* increases linearly. After 2500 h these trajectories start to plateau towards a minimal value for *P* and a maximal value for *S*. Here the model allows us to extrapolate the trajectories beyond the end of the experiment where, given the minimal growth rate, it would be very time consuming to collect further data. The model also predicts a peak in the proportion of growth-arrested cells at around 2200 h; see Discussion.

### 3.2. Analysis of experimental strategies for efficiently measuring senescence time-course

The experimental dataset collected for this study consisted of five markers spanning the replicative life-course at 16 timepoints from passage 5 to 20 (80 measurements total). This time-intensive approach provides a detailed description of the dynamics of each marker. Here we illustrate how the correct strategy for reducing the required number of measurements can be used in conjunction with the dynamical systems model to efficiently reproduce the trajectory of the markers and of the populations (*P*, *S*, *G*, *A*).

**Figure 4.**
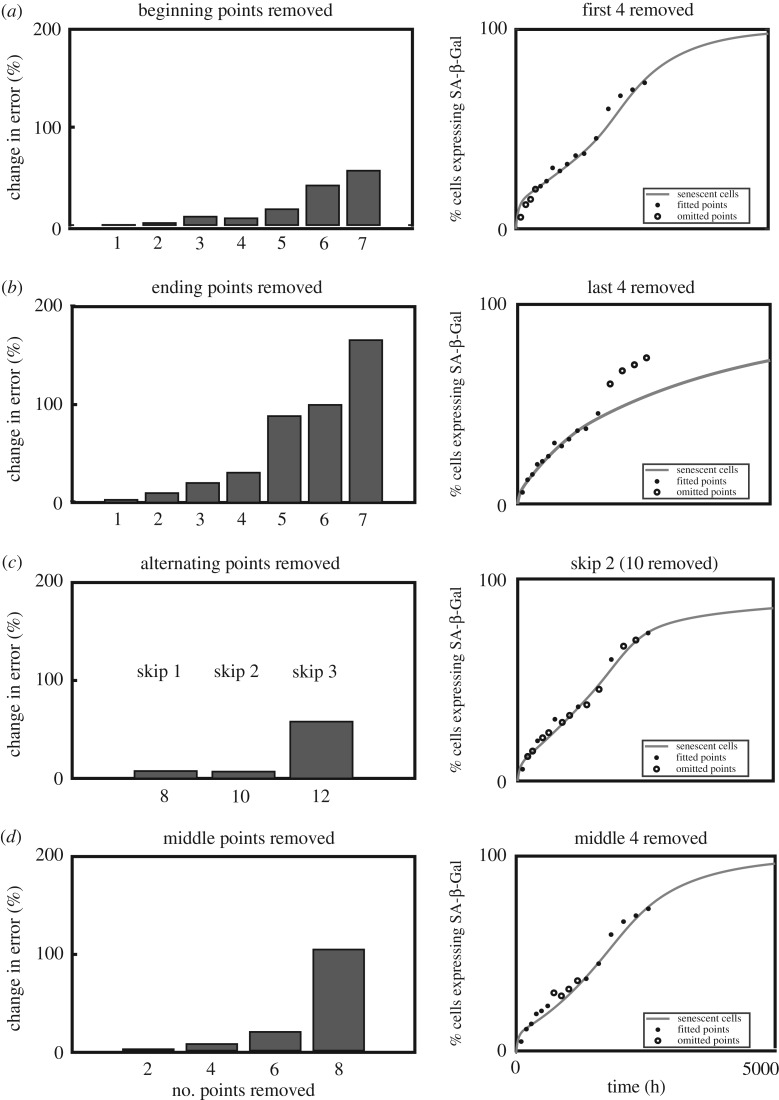
Analysis of strategies for reconstructing the senescence time-course using fewer recordings. (*a–d*) Each row illustrates a different strategy for collecting less data. Left panels show relative increase in error when trajectories are constructed as in figure 1 but using fewer data points in the parameter optimization. The baseline is the cost function error if all data points are included. Right panels show an example of the fit for SA-β-Gal when quoted number of points are removed (open circles show omitted points). (*a*) Removing data points at the beginning of the experiment quickly has a large effect on the overall error. Note that while SA-β-Gal may be still reasonably well tracked by the model output, the fit for other markers is worse (data not shown). (*b*) Removing data points from the end of the experiment has a dramatic effect when more than four passages are omitted. (*c*) Removing intermediate data points has a relatively small effect on the error introduced. (*d*) Removing intermediate blocks of points has a relatively small effect on the error introduced but there is a dramatic increase for more than 6 points.

Figure 4 shows, taking the full set of 80 measurements (5 markers, 16 points) as the baseline, how much error is introduced by removing different numbers of measurements of all the markers. The error is computed as percentage increase in the optimization cost function relative to the baseline value (see Methodology); one can think this increase in error as a proxy for the amount of information lost by not making measurements at different stages of the experiment. Different strategies can be employed: not measuring early passages (figure 4*a*), not measuring the late passages (figure 4*b*), not measuring intermediate passages (figure 4*c*) or not measuring a set of points from the middle of the experiment (figure 4*d*). If 1–2 points are removed from the data, irrespective of when during the experiment, this has little impact on the error (see figure 4*a*,*b*,*d*). However, once more than 4 points are removed the strategy is important, with data from the final passages being more valuable (compare figure 4*b* with 4*a* and figure 4*d*). The best strategy is to remove intermittent points, i.e. to take regularly spaced measurements over the course of the entire experiment (compare figure 4*c* with other panels). These results show that skipping 2/3 measurements (‘skip 2’) introduces around 10% error while reducing the number of measurements from 80 to 30 (10 measurements skipped for each of the five markers).

Our results demonstrate an optimal strategy for reducing the number of experimental measurements taken. Taking equally spaced measurements throughout proved to be a good strategy to minimize the effective loss of information, rather than omitting measurements at the beginning, end or consecutively in the middle of an experiment. The worst strategy proved to be omitting measurements at the end of the experiment, even though in practical terms these may take the longest to obtain as the growth rate slows. Notably, experimental strategies predicted by the model assume that an initial seed population (consisting entirely of proliferative cells) is allowed to transition to senescence uninterrupted. For example, in its current form, this model cannot account for the substantial metabolic and biochemical alterations likely to occur following freeze–thaw.

### 3.3. Model optimization sensitivity analysis

**Figure 5.**
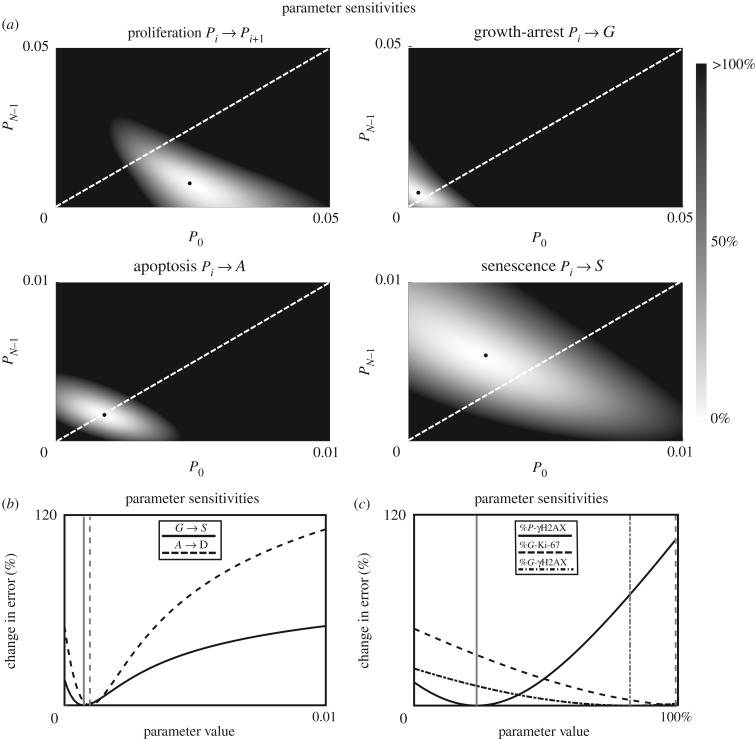
Optimization sensitivity analysis for individual parameters. (*a*) Increase in error (grey scale map) for changes to transition rate parameters relative to the optimized values (black dots). Two-dimensional plots show the value of the indicated rate parameter initially, at *P*_0_ (*x*-axis) and for the oldest proliferating cells, $P_{N-1}$ (*y*-axis). Values along the diagonal (dashed white) indicate a flat relationship (no change in rate with doubling age). Values below (above) the diagonal indicate a decreasing (increasing) relationship with doubling age. Compare best-fit points (black dots) with slope of curves in figure 1*b*. (*b*) Sensitivity to rates of growth-arrested to senescent and apoptotic to dead states, which were assumed not to depend on doubling age (vertical lines are best-fit values). (*c*) Sensitivity to marker-proportion parameters.

A sensitivity analysis was performed to assess the relative importance of different parameters for the model optimization. Figure 5*a*,*b* illustrates the relative increase in error (relative to the optimized solution, a black dot in each panel) for changes to model parameters controlling the rates of the transitions between different states, as depicted by arrows in figure 1*a*. Where these rates were assumed to depend on doubling age, a two-dimensional plot shows the initial rate at *P*_0_ versus the final rate at $P_{N-1}$ with the change in error as a greyscale map; see figure 5*a*. For the rate of transitions between proliferating populations, a decreasing relationship was optimal (i.e. the growth rate slows with doubling age). Note there would be a significant (around 50%) increase in the error if this relationship were assumed flat (along the dashed white line). For the rates of transition from proliferating to growth-arrested and from proliferating to senescent, the best-fit solutions corresponded to increasing relationships with doubling age (see slopes in figure 1*a*); however, there would not be a dramatic increase in error if these were considered constant, i.e. took values along the diagonal. For the rate of transition from proliferative to apoptotic the best-fit solution corresponded to a flat relationship (no change with doubling age) and the error would increase rapidly if this were not the case. Figure 5*b* shows that the error would increase quite rapidly if the rates of transition from grow-arrested to senescent were to increase. However, if the rate of growth-arrested to senescent was to be reduced to 0, this would not result in a particularly large increase in error, suggesting a possible simplification to the model.

A further analysis using the *holdout method* was performed to assess the effect on the additional error introduced when omitting some experimental data from the optimization (see electronic supplementary material for full details). Overall we found a small (2%) increase in the total error introduced when removing around 20% of the data points before optimization. Furthermore, statistical tests to see whether fixing any single parameter during optimization reduced the error introduced by holding out data did not reach significance. These results suggest that the original optimization approach effectively found a global minimum and that redundancy for the parameters used for optimization is low.

The final three parameters shown in figure 5*c* determine the proportion of cells in each population that contribute towards the model's representation of γH2AX and Ki-67. Rather than setting arbitrary values for these proportions, they were left as free parameters in the optimization (as there is little available evidence to fix them). Importantly, this sensitivity analysis shows that effectiveness of the optimization only depends weakly on these parameters; see Discussion as to whether these values might generalize across cell models.

## 4. Discussion

We present here the first model capable of combining multiple experimental markers to reproduce the complete time-course of a cell population ageing towards replicative senescence. The model provides a full account of the proportion of cells in each of four different states (proliferating, senescent, growth-arrested and apoptotic) and predicts the convergence of these proportions beyond the end of the experiment. The close match to our experimental data was found to be robust with respect to small changes in parameter values. The model description is parsimonious, defined in terms of a minimal set of parameters that are well constrained by the experimental data. Our investigation of different strategies for collecting data more sparsely shows that similar results could be attained by collecting as few as 30 measurements (taking sparse measurements, equally spaced by passage). Indeed, the model could provide a good means to predict when to stop making measurements, thus allowing for better planning of commitments to bench time. The modelling framework has great potential as a tool for the senescence and ageing research communities.

Various modelling approaches have been used to investigate senescence. Lawless *et al*. [29] modelled proliferating and senescent populations using differential equations (a dynamical system) and were able to reproduce some of the characteristics of experimental markers such as population doublings and SA-β-Gal (similarly SAHF) but not of other markers such as γH2AX and Ki-67. The dynamical systems model presented here can be viewed as a significant advance on the earlier model presented in [29]. Notably, the model presented here includes the doubling age of the proliferating populations (see distinct *P* populations in figure 1*a*). This allows for the rates of transition from the proliferating population to depend on doubling age (figure 1*b*). Further, by considering not only the senescent and proliferating population, as in [29], but also the growth-arrested and apoptotic populations, we were able to give a complete picture of the dynamics and to obtain a good match between model outputs and all the markers recorded. Multiple proliferative populations have been considered in models geared towards studying haematopoietic stem cells [30] and cancer [31,32]. Spatial interactions in two dimensions have also been considered in tumour models [33], a significant increase in complexity relative to the approach used here. In the present study the dynamics of the growing cell population and the transitions from proliferation were assumed to be deterministic at the level of the entire population. Other studies have considered probabilistic descriptions of the transitions between cell states [34] as influenced by cell signalling factors. A stochastic model has been used to explore observed increases in reactive oxygen species concentration with age [35]. Another model was defined at a more granular level—investigating gene signalling networks [36]; however, the model has over 60 parameters and 14 state variables, meaning that it is unlikely that the model is meaningfully constrained by available experimental data.

The structure of the model presented is general and, although used here to fit data from a monolayer human primary fibroblast culture, it could fit data from other cell types using similar markers that provide sufficient information about each cell subtype (proliferating, senescent, growth-arrested and apoptotic) with little modification. Differences between an artificial experimental system and *in vivo* measurements could be explored in further studies. Validation of the approach with other cell types remains to be done and this would be a necessary step to demonstrate the generalizability of the framework. Incorporating different markers for use with the model requires that the relative contribution of each population to the marker be proposed. As was done in this study for γH2AX and Ki-67, some of these contributions could be left unspecified (left as free parameters in this study, these contributions did not have a strong bearing on the accuracy of the model fit to data; figure 5). Furthermore, while there may be significant differences across cell types in terms of the strength of the paracrine ‘bystander’ effect induced by SASP which may influence the point at which a culture becomes static [37], the modelling framework presented here is adaptable to take these differences into account. The use of a similar model and marker set in different cell types could allow establishment of which parameters are cell-type dependent and which remain fixed. In the light of these findings, it may be possible to use fewer markers to constrain the model and accurately reproduce the senescence time-course. We note that the number of cells entering apoptosis in each passage appears to be consistently around 5–10% here and in our previous experimental work [14]. Furthermore the model could help characterize these differences across cell types in terms of biologically meaningful parameters. A distinction between senescent and growth arrest [21] is necessary to account for the markers measured in our experiments. While making a distinction between senescent and growth-arrested cells in the model, it was assumed that the reversal of growth arrest back into a proliferative state was not significant. The assumption is based on the observation that factors (SASP released from senescent cells) inducing growth arrest cannot recede in the experiments performed here. We note that a peak in the number of growth-arrested cells is predicted by the model, its presence being reliant on the presence of transitions from growth-arrested to senescent as proposed in [38]. The effect of removing transitions from growth-arrested to senescent is discussed in the electronic supplementary material, figure S1. Transitions from growth arrest to apoptotic were not considered as these cells would not be going through the cell cycle [39]. Additional analysis showed that changing the rate of transitions to the apoptotic and growth-arrested populations had a much smaller effect on the time to reach 85% senescence in comparison with changing the proliferation rate or rate of transitions to senescence (see electronic supplementary material, figure S2). A significant simplification of the present model is that no distinction is made between different stages of the cell cycle and that growth-arrest or senescence can occur in different ways [40]. Cells arrested in G1 would be predicted to stain positively for SA-β-Gal and γH2AX, but not for Ki-67, whereas cells in G2 would stain positively for all three markers.

A dynamical systems approach has proven an effective strategy for modelling the proliferation and progression towards senescence of cultured cells. The presented model goes beyond previous studies in being able to capture the trajectory of multiple markers simultaneously while giving a complete account of the cell population's different subtypes. The potential to generalize the adaptable framework presented should be tested in future work using data collected from other cell types and markers. An extension to consider further information through the cell cycle—only mitosis was considered here—would allow for a more detailed link to experimental markers. Here we provide a new approach to the study of ageing, developing a dynamical systems model that offers a complete accurate description of the dynamics of the transition to cellular senescence at the population level. Our model highlights the utility of predictive mathematical modelling in better understanding the factors of cellular senescence and the ageing process itself.

## Supplementary Material

Model equations

## Data accessibility

All experimental data and source material required to reproduce our modelling results (Matlab code) are available at the following GitHub repository: https://github.com/dgalvis/Senescence_Model.
