# Supplementary material for: A dynamical systems model for the measurement of cellular senescence
Source: J R Soc Interface. 2019 Oct 9;16(159):20190311. doi: 10.1098/rsif.2019.0311 (PMC6833332; doi:10.1098/rsif.2019.0311)
Supplement: Model equations [file rsif20190311supp1.pdf]

Nomenclature

Proliferative populations (P_i for $i=0\ldots N-1$, $P=\sum_{i=0}^{N-1} P_i$)

Growth-arrested population (G)

Apoptotic population (A)

Senescent population (P_N/S)

Dead (not a population/removed from the system; D)

Total population ($TP=P+G+S+A$)

Arbitrary Model Population (X)

Total population doublings (PD)

Rates (m)

Markers (PD, SA-β-Gal, γH2AX, Ki-67, TUNEL. Y denotes an arbitrary marker)

Number of Passages (M)

Model equations

The model is defined by the following system of ordinary differential equations (refer to Fig 1 in the manuscript for a schematic diagram of the possible transitions between populations):

$$\frac{dP_0}{dt}=-\left(m_{P_0P_1}+m_{P_0S}+m_{P_0A}+m_{P_0G}\right)P_0$$

$$\frac{dP_i}{dt}=2m_{P_{i-1}P_i}P_{i-1}-\left(m_{P_iP_{i+1}}+m_{P_iS}+m_{P_iA}+m_{P_iG}\right)P_i \quad \text{for } i=1\ldots N-1$$

$$\frac{dS}{dt}=\frac{dP_N}{dt}=2m_{P_{N-1}P_N}P_{N-1}+\sum_{i=0}^{N-1} m_{P_iS}P_i$$

$$\frac{dG}{dt}=-m_{GS}G+\sum_{i=0}^{N-1} m_{P_iG}P_i$$

$$\frac{dA}{dt}=-m_{AD}A+\sum_{i=0}^{N-1} m_{P_iA}P_i$$

Note: $m_{P_{N-1}P_N}$ denotes cells that go through the maximum number of divisions. They are then senescent. $m_{P_{N-1}S}$denotes cells that jump to senescence when they could still divide one more time.

Note: $m_{X_1X_2}$ is the rate of transition from population X_1 to population X_2.

Data Fit/Cost Function

In the optimisation routines the following cost function was used:

$$Error = \sqrt{\sum_{Y} \sum_{i=1}^{M} \left(\frac{X_{data_i} - X_{model_i}}{\max_j X_{data_j}} \right)^2} ,$$

where $M = 16$ is the total number of passages in the experiment and i is an index for each passage. Y is a list of the markers: PD, SA-β-Gal, Ki-67, γH2AX, and TUNEL.

PD_{data} is the total number of times the population has doubled calculated from cell counts at each passage. All of the other marker values Y_{data} are taken from counting the fraction of cells expressing the marker at each passage.

PD_{model} is derived from the increase in the sum of all populations P, G, S, A over time in the model.

SA-β-Gal_{model} is the fraction of S cells in the model population over time.

Ki-67_{model} is given by the fraction $\frac{P + G_{Ki-67} * G}{TP}$ in the model population over time where G_{Ki-67} is the fraction of the contribution of the growth-arrested population to the marker Ki-67.

γH2AX_{model} is given by the fraction of $\frac{S + A + G_{\gamma H2AX} * G + P_{\gamma H2AX} * P}{TP}$ in the model population over time where $G_{\gamma H2AX}$ is the fraction of the contribution of the growth-arrested population to the marker γH2AX and $P_{\gamma H2AX}$ is the fraction of the contribution of the proliferating population to the marker γH2AX.

$TUNEL_{model}$ is the fraction of *A* cells in the model population over time.

Note: in some cases, we optimize the cost function using a subset of the passages i (see Fig 4 in the main manuscript). In this case we define two different error functions, $Error_{¿}$ and $Error_{total}$. $Error_{¿}$ is the error from only the included passages; $Error_{total}$ is the error when all passages are included.

Change in Error

The change in error for both the re-optimizations and the sensitivity analysis are calculated as follow:

$$\% Change \in Error = \frac{100 * New\ Error - Original\ Error}{Original\ Error}$$

Parameters/Constraints

The state number *N* = 50 is the number times a cell in the population can divide (analogous to the Hayflick number). We select 50 rather than optimizing for the value because the change in the error of the cost function changes very little with respect to changes in *N*. We tried *N* = 40, 50, 60, 100, 150. The relative changes in %error are +1.271%, 0.000% (comparing *N* = 50 to *N* = 50), -0.377%, -1.101%, -0.071% respectively. The transition rate parameters m_{GS} andm_{AD} are positive numbers determined through optimisation (2 of 14 free parameters). The transition rate parameters $m_{P_i P_{i+1}}$,$m_{P_i S}$,$m_{P_i G}$,$m_{P_i A}$ are lists of N values with a linear relationship to N as

set by the positive-valued end points $m_{P_1 X}$ and $m_{P_N X}$ determined by optimisation (8 of 14 parameters). The fraction of contribution from each population (P or G) to each marker (γH2AX or Ki-67) is set by $P_{\gamma H2AX}$, G_{Ki-67}, $G_{\gamma H2AX}$, which take values between 0 and 1 are were determined by optimisation (3 of 14 free parameters). These contributions are not known but form part of the optimisation cost function (rather than part of the dynamical system).

Optimization

We optimize using the genetic algorithm ga found in MATLAB2018B. We changed the following options: $PopulationSize=2000, MaxGenerations=1300$, and used the default values otherwise. In addition, we also ran the constrained optimization function $fmincon$ using the best solution from the genetic algorithm as an initial point. This often would improve the likelihood that the solution would reach a minimum. We increased $MaxFunctionEvaluations=10000$, and used default settings otherwise.

In an effort to reach the global minimum, we reran the optimization algorithms multiple times for each method. For the initial optimization, where $N=50$ and the full dataset was included, we ran the algorithm 20 times and used the parameter set that minimized the cost function. For every other optimization, such as changing the value of N or using a subset of the passages, we ran the algorithm 10 times each with a randomly generated seed population, and used the parameter set that minimized the cost function.

Note: In the case where we were optimizing with a subset of passages, we used the parameter set that minimized $Erro\,r_{¿}$, not $Erro\,r_{total}$. In some cases, improving the optimization of $Erro\,r_{¿}$ increased $Erro\,r_{total}$, e.g. when only fitting to a small subset of the data, say the first few points.

Sensitivity Analysis

For the sensitivity analysis (Fig 5 in the main manuscript), we changed each parameter while keeping all of the other parameters fixed at the optimized solution. In the case of parameters $m_{P_i X}$, we varied both $m_{P_0 X}$ and $m_{P_{N-1} X}$ to create 2-dimensional plots of the change in error.

Parameters	$m_{P_0 P_1}$	$m_{P_{N-1} P_N}$	$m_{P_0 G}$	$m_{P_{N-1} G}$	$m_{P_0 S}$	$m_{P_{N-1} S}$	$m_{P_0 A}$	$m_{P_{N-1} A}$	m_{GS}	m_{AD}
Rates h^{-1}	0.0276	0.0049	0.0035	0.0010	0.0028	0.0053	0.0019	0.0016	0/fixed	0.0015
Parameters	$P_{\gamma H2AX}$	G_{Ki-67}	$G_{\gamma H2AX}$							
Proportions	0.2060	0.9995	0.9533							
Parameters	N									
Value	50									

Optimization assuming $m_{GS}=0$

We investigated the case where growth-arrested cells cannot transition to senescence, resulting in an significant proportion of growth-arrested cells at the end of the experiment (See Fig S1). Comparing the table of parameters to the original parameter set (Table 1 in the manuscript), it looks like the results are similar, other than that $m_{P_0 G} > m_{P_{N-1} G}$ here. Interestingly, both versions of the model suggest that

$G_{Ki-67} \approx 1$, where as $G_{\gamma H2AX}$ is closer to 1 than in the original model. We cannot draw a strong conclusion on whether the transition from G -> S should be included in the model based on the fit to experimental data. Fig S1, ~20% of cells are growth-arrested at the end and ~80% are senescent, whilst, $Error_{total}$ only increases to 0.8133 from 0.8101 in the original version of the model.

Parameters affecting time to 85% senescence

The time it takes to reach 85% senescence was investigated with the model when changing the rate parameters $m_{P_i P_{i+1}}$, $m_{P_i S}$, $m_{P_i G}$, and, $m_{P_i A}$ as shown in Fig S2. The figure shows a greyscale map of the change in time to reach 85% senescence depending on the gradient of $m_{P_i P_{i+1}}$, $m_{P_i S}$, $m_{P_i G}$, and, $m_{P_i A}$ with respect to increasing i. Larger values on the x-axis indicate a larger initial rate m and larger values on the y-axis a larger final rate m. Black dots indicate the operating values of these parameters for the model. Darker shading indicate that the progression towards senescence is slowed. The transition rates $m_{P_i P_{i+1}}$(top left panel) and $m_{P_i S}$ (bottom right panel) have the greatest effect. As can be expected, decreasing the rate of transition from proliferating to senescent does indeed slow the time to reach 85% senescence. Interestingly, slowing early and increasing later proliferation rates can decrease the rate of transition to senescence.

Parameter sensitivity with holdout method

As a further evaluation of the robustness of the model and the relative importance of each parameter, we used the holdout method of cross validation. First, we randomly selected 20 distinct sets of 13 (out of 16) time points of the data (~80% of the data). We reran the optimization on each of these testing sets (repeating each optimization 10 times as described above) to obtain a distribution of errors across testing sets. Note: as with the other subset optimizations, we optimize using $Error_{¿}$. The $Error_{total}$ was then used to calculate a distribution for the percent change in error. Moreover, for this method we also define the error of the points that were omitted from the optimization $Error_{ho}$ and the difference in error for the holdout points relative to the baseline error for the same points: $Error_{ho-baseline}$. We also found the distribution of percent change in holdout error, which is the defined as:

$$\% Change \in Holdout\ Error = \frac{100 * Error_{ho} - Error_{ho-baseline}}{Error_{h-o-baseline}}.$$

This first results from this analysis is that the mean±std changes in error over the 20 holdout re-optimizations was $Error_{total} = ¿$1.82±1.31% across all markers and data points, thus demonstrating that in general the optimization is not sensitive to the arbitrary removal of a small percentage of the data used for fitting. Specifically, for the holdout data points the mean±std error was $Error_{ho} = ¿$15.75±9.23% with the largest contribution occurring for data points at the beginning or end of the experiments (see also Fig 4 in the main paper).

As another test of parameter sensitivity, we fixed each parameter individually at the best-fit parameter from the full optimization, and reran the optimization on the same

20 sets described above, but with all the other parameters free (table below). This resulted in a % holdout error mean/std for each parameter fixed in isolation. One-sided t-tests were used testing for significant decreases in the holdout error for fixed parameters vs. all parameters free. Decreases in this error imply that fixing a given parameter limits the error introduced when points are held out.

Parameters	$m_{P_0P_1}$	$m_{P_{N-1}P_N}$	m_{P_0G}	$m_{P_{N-1}G}$	m_{P_0S}	$m_{P_{N-1}S}$	m_{P_0A}	$m_{P_{N-1}A}$	m_{GS}	m_{AD}
Mean % change holdout error	16.78	16.85	16.49	15.37	15.71	14.39	15.53	14.09	16.51	13.94
STD % change holdout error	10.77	10.88	10.22	8.31	9.25	6.70	9.67	8.70	9.84	8.23
P-value for decrease vs. baseline	0.63	0.63	0.59	0.45	0.49	0.30	0.47	0.28	0.60	0.26
Parameters	$P_{\gamma H2AX}$	G_{Ki-67}	$G_{\gamma H2AX}$	baseline						
Mean % change holdout error	15.58	15.29	16.14	15.75						
STD % change holdout error	9.69	8.91	10.44	9.23						
P-value for decrease vs. baseline	0.48	0.44	0.55	N/A						

No parameter reached significance for a reduction in error suggesting that the original optimisation approach robustly reached a global minimum and that no parameter was redundant for the optimisation.
